# Prevalence and determinants of psychological insulin resistance among type 2 diabetic patients in Kinshasa, Democratic Republic of Congo

**DOI:** 10.4102/phcfm.v11i1.1993

**Published:** 2019-07-22

**Authors:** Shomba L. Rita, Fina J.-P. Lubaki, Lepira F. Bompeka, Gboyega A. Ogunbanjo, Lukanu P. Ngwala

**Affiliations:** 1 Centre Hospitalier Mère-Enfant Monkole, Kinshasa, The Democratic Republic of the Congo; 2 Department of Family Medicine and Primary Health Care, Protestant University of Congo, Kinshasa, The Democratic Republic of the Congo; 3 Service de Néphrologie, Cliniques Universitaires de Kinshasa, Kinshasa, The Democratic Republic of the Congo; 4 Department of Family Medicine, Sefako Makgato University, Pretoria, South Africa

**Keywords:** psychological insulin resistance, therapy, type 2 diabetes mellitus, frequency, determinants

## Abstract

**Background:**

Psychological insulin resistance (PIR) is a common but unappreciated phenomenon by health care providers with a negative impact on the control of type 2 diabetes mellitus.

**Aim:**

To determine the frequency of PIR and its determinants in patients with type 2 diabetes.

**Setting:**

This study was conducted in Kinshasa in three health centres providing management of diabetic patients.

**Methods:**

This study was a multicentric, cross-sectional study conducted from 01 November 2017 to 31 March 2018 in Kinshasa among 213 type 2 diabetic patients who were taking oral anti-diabetic drugs. A standardised questionnaire, the Chinese Attitudes to Starting Insulin Questionnaire (Ch-ASIQ), was used for data collection.

**Results:**

The average age of participants was 59.8 ± 11.1 years with a male to female ratio of 1.5. The prevalence of PIR was 42.7%; and its main determinants were 50 years of age (odds ratios [OR] adjusted 2.05; 95% confidence interval [CI] 1.98–4.27; *p* = 0.045), the presence of complications (OR adjusted 3.33; 95% CI 1.68–6.60; *p* = 0.001), lack of knowledge about insulin therapy (OR adjusted 1.96; 95% CI 1.03–3.71; *p* = 0.040) and the high cost of insulin (OR adjusted 2.32; 95% CI 1.08–4.95; *p* = 0.030).

**Conclusion:**

The study showed that almost half of type 2 diabetic patients had PIR with the main determinant factors related to the patient and the health system. The establishment of a therapeutic education programme, improved ‘provider–patient’ communication and the development of approaches to increase access to drugs are crucial to reduce the prevalence of PIR.

## Introduction

The prevalence of type 2 diabetes mellitus is increasing exponentially worldwide. Particularly, low- and middle-income countries will carry a heavy burden in the next few years if there are no efforts made to prevent and ensure early detection and management of type 2 diabetes.^1^ In recent years, significant progress has been made, particularly in the development of new oral medications. However, the use of insulin remains essential at the evolutionary stage of the disease, because insulin resistance which is a characteristic of type 2 diabetes, leads over time to the depletion of beta cells of islets of Langerhans.^2^ In the United Kingdom Prospective Diabetes Study (UKPDS) study,^3^ less than 25% of normal insulin secretion was observed 6 years after diagnosis of the disease. Many patients with type 2 diabetes, who should benefit from insulin therapy during the course of their illness or in special circumstances, do not receive it at all or receive it outside of the required timing because of resistance.^2^ Psychological insulin resistance (PIR) can be explained by factors related to the patient, the provider and the health system.^2,4,5,6^ Attitudes contributing to PIR include beliefs that receiving insulin is associated with an adverse outcome and/or outcome of the disease because of non-compliance by the patient, a restriction in lifestyle habits and stigma unfavourable to glycaemic control.^2^ Management guidelines, not recommending insulin therapy unless other strategies have failed to achieve glycaemic control, also contribute to this resistance to insulin therapy from health care providers.^2^ The factors leading to PIR vary in different settings.^2^

In the Democratic Republic of the Congo (DRC), the prevalence of diabetes mellitus is estimated at 4.8%^7^ with type 2 diabetes causing high morbidity and mortality, as well as several acute conditions, such as infections, hyperosmolarity and surgical complications, requiring a switch to insulin therapy.^8,9,10,11^

The purpose of this study was to determine the prevalence of PIR in type 2 diabetic patients and its determinants in hospital settings in Kinshasa.

## Methods

### Study design

Our study was a cross-sectional study.

### Study period and setting

The study was conducted in Kinshasa from November 2017 to March 2018, in three health facilities providing care to diabetic patients: specifically, the ^1^Centre Hospitalier Mère-Enfant (CHME) Monkole Diabetology Unit located in the Mont-Ngafula 1 Health Zone, the Diabetic Clinic of the Tatamena Health Center located in the Bumbu Health Zone and the Diabetes Unit of the Strategos Medical Services (SMS) Center DRC located in the Gombe Health Zone. These health facilities organise outpatient consultations of diabetic patients at a weekly rate. At each medical consultation, patients receive training on diabetes and are examined for adjustments in their treatments according to the progression of the disease.

### Study population

All diabetic patients followed at the CHME Monkole Diabetes Unit, the Tatamena Health Center and the DRC SMS Hospital Centre during the study period represented the study population.

### Selection criteria

Patients were included in the study based on the following criteria:

- be a type 2 diabetic patient (any diabetic patient who had knowledge of the type of diabetes or had started diabetes after age 40)
- be on oral anti-diabetic medication
- be regularly followed in one of the clinics selected for the study
- give informed consent.

### Sample size and selection of the participants

The size of our sample was 213, which was calculated according to the formula: *n* ≥ (Z^2^*p* (1-*p*))/*d *^2^, with *p* = 0.5 as the prevalence of psychological insulin is unknown in our setting, *Z* = 1.96 and *d* = 0.1. As we expected a non-response rate of 10%, the minimum sample size was 105.6. In the field, we had 213 patients, representing more than twice the calculated sample size.

The participants were selected by convenience until the total of 213 patients was reached.

### Data collection

The data were collected using interviews by two investigators, the principal investigator and the co-investigator. The collection tools were a data collection sheet and the ‘Chinese Attitudes to Starting Insulin Questionnaire’ (Ch-ASIQ). The collection sheet identified socio-demographic characteristics (age, sex, educational level, marital status, occupation, income, distance to the health centre (from home) and number of persons in charge), characteristics of diabetes (duration, current treatment, existence of complication, previous hospitalization and presence of comorbidity) and knowledge about insulin therapy (insulin type, injection site, indications, side effects, storage and administration). Insulin therapy knowledge was defined from eight questions; a good knowledge was defined in all patients with a score ≥ 5 and poor knowledge was defined in all patients with a score between 0 and 4. The Ch-ASIQ is the first validated psychometric questionnaire to evaluate psychological resistance to the initiation of insulin for diabetic patients in the context of primary health care.^12^ It includes 13 items divided into four factors: self-image and stigma, factors promoting self-efficacy, fear of pain or needles, and family support. Responses to each item were coded according to a 4-point Likert scale (total disapproval = 1, disapproval = 2, approval = 3, total acceptance = 4). High scores for ‘time and family support’ and ‘self-efficiency’ factors reflect a more positive attitude to insulin therapy; high scores for ‘self-image and stigma’ and ‘fear of pain or needles’ indicate negative attitudes towards insulin initiation. All factors and total score ranged from 1 to 4 with a central point of 2.5.^12^ Psychological insulin resistance was defined in any patient with an overall score between 0 and 2, and the acceptance of insulin therapy was defined in patients with an overall score between 3 and 4. The Ch-ASIQ has an alpha coefficient of Cronbach’s alpha for all four factors that are greater than 0.60 (0.62–0.80). The questionnaire, translated from English into French and Lingala, was administered in one of the two local languages in which the patient was more comfortable speaking.

### Measures of interest for the study

The primary variable of interest was the prevalence of PIR. The secondary variable of interest was the determinants of insulin resistance.

### Statistical analysis

The data were encoded into an Excel database after cleaning and exporting to the SPSS (Statistical Package for Social Sciences software version 21, Chicago, United States). The statistical data analysis consisted of determining average, medians, interquartile range (IQR) and standard deviation for continuous variables (quantitative) and proportions of categorical variables (qualitative). The results are presented as graphs and tables. The Pearson chi-square test or the Fischer’s exact test was used to compare proportions and Student’s *t*-test and Mann–Whitney U test were, respectively, used to compare the means or medians. The determinants of knowledge and PIR were assessed through the logistic regression; the odds ratios (ORs) and their 95% confidence interval (CI) were used to estimate the strength of association between the independent and dependent variables. The value of *p* < 0.05 was the statistical significance threshold.

### Ethical considerations

The study obtained the approval of the Ethics Committee of the Protestant University of Congo (Register number CEUPC0031).

## Results

Two hundred and thirteen patients with type 2 diabetes took part in this study, and among them were 84 men and 129 women, with a sex ratio of 1:5. The average age was 59.8 ± 11.1 years with extremes of 30 and 84 years. The proportion of patients who were married, had secondary school level and unemployed was, respectively, 75.1%, 70.9% and 70%. The average monthly income was 387.3 ± 52 USD, and the median number of people in charge of diabetic patients was four. The median duration of diabetes mellitus was 4 years in 65.7% of patients; diabetes was associated with hypertension in 65.7% of patients; 34.3% of patients had a complication of diabetes; and the most common complication was peripheral neuropathy (24.4%). Fifty percent of patients had good knowledge of insulin therapy, and the cost of insulin therapy was estimated to be unaffordable by 75.1% of patients (Table 1).

**TABLE 1:** Participants general characteristics and psychological insulin resistance, Kinshasa, 2017–2018.

Variable	Total (*N* = 213)		Resistant (*N* = 91)		Non-resistant (*N* = 122)		*p*
	*N*	%	*N*	%	*N*	%	
**Age (years)**							
< 50	44	20.7	23	25.3	21	17.2	0.010
≥ 50	169	79.3	68	74.7	101	82.8	
**Sex**							
Men	84	39.4	33	36.3	51	41.8	0.025
Women	129	60.6	58	63.7	71	58.2	
**Marital status**							
Married	160	75.1	70	76.9	90	73.8	0.358
Single	53	24.9	21	23.1	32	26.2	
**Educational level**							
Other	151	70.9	61	67.0	90	73.8	0.179
College/university	62	29.1	30	33.0	32	26.2	
**Employment status**							
Unemployed	149	70.0	67	73.6	82	67.2	0.195
Employed	64	30.0	24	26.4	40	32.8	
**Time since diabetes mellitus diagnosis (years)**							
≤ 10 years	169	79.3	70	76.9	99	81.1	0.297
> 10 years	44	20.7	21	23.1	23	18.9	
Previous hospitalization	62	29.1	23	25.3	39	32.0	0.181
Complications of diabetes mellitus	73	34.3	45	49.5	28	23.0	< 0.001
Comorbidity	140	65.7	58	63.7	82	67.2	0.350
Knowledge of side effects	35	16.4	19	20.9	16	13.1	0.009
Weight gain	30	14.1	15	16.5	15	12.3	0.250
Hypoglycaemia	157	73.7	67	73.6	90	73.8	0.552
Blurred vision	8	3.8	6	6.6	2	1.6	0.005
No knowledge of insulin therapy	105	49.3	62	68.1	43	35.2	0.025
Unaffordable price	160	75.1	78	85.7	82	67.2	0.001

*N*, number.

**FIGURE 1:**
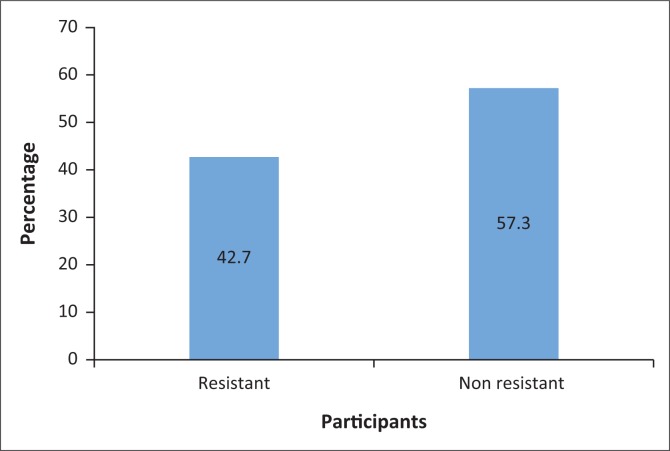
Prevalence of resistance versus acceptance of insulin among participants, Kinshasa, 2017–2018, *N* = 213.

The PIR was present in 91 participants (42.7%) (Figure 1), with 33 men (15.5%) and 58 women (27.2%) (Table 1).

Table 1 compares the general characteristics of resistant to non-resistant patients. The age up to 50 years, female gender, presence of complications of diabetes mellitus, knowledge of side effects, blurred vision, lack of knowledge about insulin therapy and the expensive cost of insulin therapy were the main variables significantly (*p* < 0.001) associated with PIR compared to non-resistant patients.

In the multivariate analysis (Table 2), age ≤ 50 years (OR adjusted 2.05; 95% CI [1.98–4.27]; *p* = 0.045), the presence of complications (OR adjusted 3.33; 95% CI [1.68–6.60]; *p* = 0.001), lack of knowledge on insulin therapy (OR adjusted 1.96; 95% CI [1.08–3.71], *p* = 0.040) and the unaffordable cost of insulin therapy (OR adjusted 2.32; 95% CI [1.08–4.95]; *p* = 0.030) had emerged as the main independent determinants of PIR.

**TABLE 2:** Determinants of resistance to insulin therapy in multivariate analysis.

Variable	Univariate			Multivariate		
	*p*	OR	CI 95%	*p*	OR adjusted	CI 95%
**Age (year)**						
≥ 50	-	1.00	-	-	1.00	-
< 50	0.015	1.63	1.38–3.17	0.045	2.05	1.98–4.27
**Sex**						
Male	-	1.00	-	-	1.00	-
Female	0.041	1.62	1.72–2.21	0.184	1.53	0.82–2.89
**DM complication**						
No	-	1.00	-	-	1.00	-
Yes	<0.001	3.28	1.82–5.92	0.001	3.33	1.68–6.60
**Knowledge of side effects**						
No	-	1.00	-	-	1.00	-
Yes	0.013	1.75	1.84–3.63	0.683	1.22	0.48–3.12
**Blurred vision**						
No	-	1.00	-	-	1.00	-
Yes	0.018	4.24	1.18–9.49	0.319	2.74	0.38–9.77
**Knowledge of insulin therapy**						
Yes	-	1.00	-	-	1.00	-
No	0.004	2.25	1.72–3.46	0.04	1.96	1.03–3.71
**TTT cost**						
Affordable	-	1.00	-	-	1.00	-
Unaffordable	0.003	2.93	1.46–5.88	0.03	2.32	1.08–4.95

DM, diabetes mellitus; TTT, treatment; OR, odds ratio; CI, confidence interval.

## Discussion

In our study, almost half of type 2 diabetic patients had PIR with independent determinants such as age < 50 years, presence of complications, lack of knowledge about insulin therapy and low cost of insulin.

The prevalence of PIR in our study was close to the 42%, 40% and 47.2% reported in the studies conducted, respectively, in Bangladesh,^13^ the United States^14^ and Egypt.^15^ This prevalence is lower than 51%, 53.29%, 70.6% and 72% found in Asia.^16,17,18,19^ On the other hand, it is higher than 27%, 28% and 33% reported by European authors.^3,20,21^ This high prevalence in our study may be because of a weak integration of diabetes care at the primary care level, misinformation and lack of training of diabetic patients on diabetes, its complications and the benefits of insulin therapy. Differences in the age of patients included in the various studies may also explain this disparity in PIR frequency.^22^ Low knowledge of diabetes can negatively affect beliefs and perceptions about insulin therapy as demonstrated by other authors who have evaluated PIR.^22^ Being under 50 years of age has been one of the determinants of PIR in our study. Age as a determinant has not been found by other authors.^14^ The diverse population studied, the method used and the sample size can explain this difference. Machinani et al. conducted their study on different ethnic groups (African Americans and Latin Americans),^14^ whereas our study focused on a homogenous population in terms of race with participants living in the same socio-economic and cultural context. Restrictions and the adaptation of lifestyle habits are all factors that may explain the prevalence of PIR in patients under 50 years of age.

Nearly half of the patients had diabetes complications that were associated with a negative attitude towards insulin therapy. This observation is similar to that made by Lee in China and Brod et al., in a systematic review.^22,23^ Complications, especially in a patient undergoing treatment, are experienced as an injustice and a progression that can lead to death. Lack of knowledge about insulin therapy emerged as one of the main factors associated with PIR in our study. This observation has already been made by Karter et al, who linked the lack of knowledge about diabetes to a quality problem of communication between caregiver and patient.^24^ The reasons given by the patients included, among other things, not having received information, misunderstanding the information received either because of a vision problem, or the inability to read the documents made available to them.^24^ Other studies have found that fear of initiation to insulin therapy was associated with poor beliefs: fear of injections, stigma, addiction, symptoms synonymous with complications and death, the onset of hypoglycaemia and complications.^8,25^ In the study, it was also noted that patients did not have a knowledge of insulin indications and adverse effects at injection sites. Poor perception of insulin therapy as the basis of PIR in our midst is also linked, among other things, to the low level of education of patients as also found by Jasper et al.^25^ The cost of insulin was considered unaffordable in three quarters of cases and in almost 9 out of 10 patients, respectively, in the whole group and in patients with a negative attitude towards insulin therapy. The unaffordable cost was found to be a significant barrier to insulin therapy and a factor favouring PIR in Nigeria with an average monthly cost of $39.00.^26,27,28^ The lack of state subsidies, the grouping of patients in association with diabetes and a policy of diabetes control in the face of the progress of the pharmaceutical industry are all factors in favour of the PIR. The absence of a social policy affects the entire supply and distribution circuit and increases the unit price of insulin. Government involvement in the purchase of insulin, and the choice of providers and products can improve insulin availability.^29^

## Conclusion

This study showed that almost half of the patients with type 2 diabetes had PIR with the main determinant factors related to the patient and the health system. The establishment of a therapeutic education programme on diabetes, improved ‘provider–patient’ communication and the development of approaches to geographic and financial access to essential drugs are crucial to reduce the prevalence of PIR and its deleterious consequences on the control of diabetes mellitus and the quality of life of patients.

## References

[1] Hall V, Thomsen RW, Henriksen O, et al Diabetes in sub-Saharan Africa 1999–2011: Epidemiology and public health implications. A systematic review. BMC Public Health. 2011;11:564 https://doi.org/101186/1471-2458-11-564 21756350 10.1186/1471-2458-11-564 PMC3156766

[2] Peyrot M, Rubin R, Lauritzen T, et al Resistance to insulin therapy among patients and providers: Results of the cross-national diabetes attitudes, wishes and needs (DAWN) study. Diabetes Care. 2005;28:2673–2679. 10.2337/diacare.28.11.2673 16249538

[3] UK Prospective Diabetes Study (UKPDS) Group Intensive blood-glucose control with sulphonylureas or insulin compared with conventional treatment and risk of complications in patients with type 2 diabetes (UKPDS 33). UK Prospective Diabetes Study (UKPDS) Group. Lancet. 1998 352(9131):837–853. 10.1016/S0140-6736(98)07019-6 9742976

[4] Taylor CG Jr, Taylor G, Atherley A, et al The Barbados Insulin Matters (BIM) study: Barriers to insulin therapy among a population-based sample of people with type 2 diabetes in the Caribbean island of Barbados. J Clin Transl Endocrinol. 2017;8:49–53. 10.1016/j.jcte.2017.04.002 29067259 PMC5651331

[5] Ng C, Lai P, Lee Y, et al Barriers and facilitators to starting insulin in patients with type 2 diabetes: A systematic review. Int J Clin Pract. 2015;69 (10):1050–1070. 10.1111/ijcp.12691 26147376

[6] Haque M, Navsa M, Hayden ES, et al Barriers to initiating insulin therapy in patients with type 2 diabetes mellitus in public-sector primary health care centres in Cape Town. S Afr Med J. 2005;95:798–802. 10.1080/22201009.2005.10872127 16341336

[7] IDF diabetes atlas – 8th edition [homepage on the Internet]. [cited 2018 Nov 11]. Available from: http://www.diabetesatlas.org/

[8] Bieleli EI, Moswa JL, Ditu M, et al Prévalence du diabète sucré au sein de la population de Kinshasa. Congo Méd. 2000;2(15):1058–1061.

[9] Kasiam JB, Longo-Mbenza B, Nge Okwe A, et al Prevalence, epidemic’s stage and risk factors of diabetes mellitus in Kinshasa Hinterland. Int J Diabetes Metab. 2008;16:50–59.

[10] Katchunga P, Hermans MP, Manwa B, Lepira F, Kashongwe Z, M’Buyamba-Kabangu JR Hypertension artérielle, insulinorésistance et maladie rénale chronique dans un groupe de diabétiques de type 2 du Sud-Kivu, RD Congo. Néphrol Thér. 2010;6(6):520–525. 10.1016/j.nephro.2010.04.002 20605543

[11] Makulo J, Rissassi, Nseka M, et al Albuminurie pathologique lors du dépistage du diabète en milieu semi-rural (cité de Kisantu en RD Congo). Néphrol Thér. 2010;6(6):513–519. 10.1016/j.nephro.2010.04.006 20627763

[12] Sau-Nga F, Wong CKH, Chin WY, et al Association of more negative attitude towards commencing insulin with lower glycosylated hemoglobin (HbA1c) level: A survey on insulin-naïve type 2 diabetes mellitus Chinese patient. J Diabetes Metab Disord. 2016;15:3 10.1186/s40200-016-0223-0 26913243 PMC4765059

[13] Khan H, Lasker SS, Chowdhury TA Prevalence and reasons for insulin refusal in Bangladeshi patients with poorly controlled type 2 diabetes in East London. Diabetic Med. 2008;25:1108–1111. 10.1111/j.1464-5491.2008.02538.x 19183316

[14] Machinani S, Bazargan-Hejazi S, Hsia SH Psychological insulin resistance among low-income, US racial minority patients with type 2 diabetes. Prim Care Diabetes. 2013;7:51–55. 10.1016/j.pcd.2012.11.003 23254254 PMC3593744

[15] El Shafei MM, Sayyah HS, Hussein R Psychological insulin resistance in patients with type 2 diabetes mellitus. Egypt J Psychiatr. 2015;36:60–65. 10.4103/1110-1105.153794

[16] Shah SMA, Butt Z, Hussain K Factors leading to psychological insulin resistance among patients with type 2 diabetes mellitus. Ann Pak Inst Med Sci. 2017;13(3):226–230.

[17] Nur Azmiah Z, Zulkarnain AK, Tahir A Psychological insulin resistance (PIR) among type 2 diabetes patients at public health clinics in federal territory of Malaysia. Int Med J Malaysia. 2011;10(2):7–12.

[18] Wong S, Lee J, Ko Y, et al Perceptions of insulin therapy amongst Asian patients with diabetes in Singapore. Diabet Med. 2011;28:206–211. 10.1111/j.1464-5491.2010.03195.x 21219431

[19] Yiu MP, Cheung KL, Chan KW A questionnaire study to analyze the reasons of insulin refusal of DM patients on maximum dose of oral hypoglycemic agents (OHA) among3 GOPC in Kowloon West Cluster [homepage on the Internet]. [cited 2018 Nov 11]. Available from: http://www.ha.org.hk/haconvention/hac2010/proceedings/pdf/Poster/spp-p5-38.pdf

[20] Polonsky WH, Fisher L, Guzman S, et al Psychological insulin resistance in patients with type 2 diabetes: The scope of the problem. Diabetes Care. 2005;28:2543–2545. 10.2337/diacare.28.10.2543 16186296

[21] Larkin ME, Capasso VA, Chen CL, et al Measuring psychological insulin resistance: Barriers to insulin use. Diabetes Educ. 2008;34(3):511–517. 10.1177/0145721708317869 18535324

[22] Brod M, Lessard AS, Meneghini L Psychological insulin resistance: Patient beliefs and implications for diabetes management. Qual Life Res. 2009;18;23–32. 10.1007/s11136-008-9419-1 19039679

[23] Lee KL Psycholosocial factors associated with psychological insulin resistance in primary care patients in Hong Kong. J Clin Transl Endocrinol. 2015;2(4):157–162. 10.1016/j.jcte.2015.10.001 29159120 PMC5685026

[24] Karter AJ, Subramanian U, Saha C, et al Barriers to insulin initiation: The translating research into action for diabetes insulin starts project. Diabetes Care. 2010;33(4):733–735. 10.2337/dc09-1184 20086256 PMC2845015

[25] Jasper US, Opara MC, Pyiki ED, et al Knowledge of insulin use and its determinants among Nigerian insulin requiring diabetes patients. J Diabetes Metab Disord. 2014;13:10 10.1186/2251-6581-13-10 24397956 PMC3933982

[26] Fadare J, Olamoyegun M, Gbadegesin BA Medication adherence and direct treatment cost among diabetes patients attending a tertiary healthcare facility in Ogbomosho, Nigeria. Malawi Med J. 2015;2(2):65–70. 10.4314/mmj.v27i2.7 PMC4562083 26405515

[27] Management Sciences for health International medical products price guide [homepage on the Interne]. c 2015 [cited 2018 Nov 11]. Available from: https://www.msh.org/resources/international-medical-products-price-guide.

[28] Gulam AH, Otieno CFF, Omondi-Oyoo G Prevalence of psychological insulin resistance among patients with type 2 diabetes at Kenyata National Hospital, Kenya. Health Sci J. 2017;11:3 10.21767/1791-809X.1000508

[29] Novo Nordisk Sustainability report 2003 [homepage on the Internet]. [cited 2018 Nov 11]. Available from: https://www.unglobalcompact.org/system/attachments/7580/original/NovoNordisk_Sustainability_Report_2003.pdf?1282019220

